# Intrinsic and tunable ferromagnetism in Bi_0.5_Na_0.5_TiO_3_ through CaFeO_3-δ_ modification

**DOI:** 10.1038/s41598-020-62889-w

**Published:** 2020-04-10

**Authors:** N. T. Hung, N. H. Lam, A. D. Nguyen, L. H. Bac, N. N. Trung, D. D. Dung, Y. S. Kim, N. Tsogbadrakh, T. Ochirkhuyag, D. Odkhuu

**Affiliations:** 1 School of Engineering Physics, Ha Noi University of Science and Technology, 1 Dai Co Viet road, Ha Noi, Viet Nam; 2 Department of Physics, Faculty of Basic-Fundamental Sciences, Viet Nam Maritime University, 484 Lach Tray Road, Le Chan, Hai Phong, Viet Nam; 3 Department of Physics, University of Ulsan, Ulsan, 680-749 Republic of Korea; 4 Department of Physics, National University of Mongolia, Ulaanbaatar, 14201 Mongolia; 5 Department of Physics, Incheon National University, Incheon, 22012 Republic of Korea

**Keywords:** Ferromagnetism, Magnetic properties and materials

## Abstract

New (1-*x*)Bi_0.5_Na_0.5_TiO_3_ + *x*CaFeO_3-δ_ solid solution compounds were fabricated using a sol–gel method. The CaFeO_3-*δ*_ materials were mixed into host Bi_0.5_Na_0.5_TiO_3_ materials to form a solid solution that exhibited similar crystal symmetry to those of Bi_0.5_Na_0.5_TiO_3_ phases. The random distribution of Ca and Fe cations in the Bi_0.5_Na_0.5_TiO_3_ crystals resulted in a distorted structure. The optical band gaps decreased from 3.11 eV for the pure Bi_0.5_Na_0.5_TiO_3_ samples to 2.34 eV for the 9 mol% CaFeO_3-δ_-modified Bi_0.5_Na_0.5_TiO_3_ samples. Moreover, the Bi_0.5_Na_0.5_TiO_3_ samples exhibited weak photoluminescence because of the intrinsic defects and suppressed photoluminescence with increasing CaFeO_3-δ_ concentration. Experimental and theoretical studies via density functional theory calculations showed that pure Bi_0.5_Na_0.5_TiO_3_ exhibited intrinsic ferromagnetism, which is associated with the possible presence of Bi, Na, and Ti vacancies and Ti^3+^-defect states. Further studies showed that such an induced magnetism by intrinsic defects can also be enhanced effectively with CaFeO_3-δ_ addition. This study provides a basis for understanding the role of secondary phase as a solid solution in Bi_0.5_Na_0.5_TiO_3_ to facilitate the development of lead-free ferroelectric materials.

## Introduction

The integration of room-temperature ferromagnetic behavior in lead-free ferroelectric materials is a new research trend for the development of green functional materials in smart electronic devices^1,2^. PbTiO_3_-based compounds are one of the most commonly used ferroelectric materials in electronic devices^3^. Therefore, ferroelectric PbTiO_3_-based materials with improved magnetic properties have the potential for the fabrication of next-generation electronic devices.

First, the self-organized ferromagnetism of pure ferroelectric PbTiO_3_ materials was investigated. The experimental results showed that the weak ferromagnetism in undoped PbTiO_3_ nanocrystalline at room temperature resulted from intrinsic defects in events such as O and Ti vacancies^4^. PbTiO_3_ thin films also exhibited room-temperature ferromagnetism because of defects in the crystal quality of the film during growth^5^. Shimada *et al*. predicted that both O and Ti vacancies induce ferromagnetism but through different mechanisms. The ferromagnetism driven by O vacancies originated from the spin-polarized *e*_g_ state of the nearest Ti atom, whereas that directed by Ti vacancies was attributed to the half-metallic *p*_x_ state of the nearest O atom^6^. In addition, the ferroelectric property of PbTiO_3_ materials at room temperature could be attributed to O vacancies formed on the surfaces, such as vacancies induced ferromagnetism due to local non-stoichiometry and orbital symmetry breaking^7^. Xu *et al*. conducted first-principle calculations and reported that the O vacancies that formed at the domain wall led to magnetism with a localized spin moment around the vacancies^8^. Second, the conversion of transition metals to ferroelectric PbTiO_3_ materials was studied to overcome the limitations associated with the presence of rare multiferroic materials in nature^9,10^. Fe-doped PbTiO_3_ nanocrystals were reported to exhibit room-temperature ferromagnetism^11^. The saturation magnetization of these materials was improved using polyvinyl alcohol as surfactant^12^. Oanh *et al*. reported that Mn and Ni substitution at the Ti site in PbTiO_3_ materials also exhibited room-temperature ferromagnetism^13,14^. Weston *et al*. predicted that Co-doped PbTiO_3_ is a bi-stable magnetic system with strong spin-lattice coupling, where the spin-lattice effect mediates magnetoelectric coupling and the electric field possibly induced spin-crossover^15^. Third, ferroelectric and ferromagnetic materials with magnetoelectric coupling were fabricated as a multilayer and composite, where the magnetic and electrical-fields possible controlled polarization and magnetization, respectively.

Interestingly, the magnetoelectric effect has been reported in ferromagnetic grains that are distributed randomly in host ferroelectric materials as composites and/or ferromagnetic thin films grown on ferroelectric and multilayered ferroelectric/ferromagnetic thin films^16–18^. Recent developments in the ferromagnetic properties of ferroelectric PbTiO_3_-based materials have allowed the fabrication of new materials for the next-generation technologies. The application of PbTiO_3_-based material is limited mainly by the contamination risk of Pb, which accounts for more than 60 wt.% of the material, possibly leading to pollution and harmful effects on human health. Hence, there is a strong need for green ferroelectric materials to replace Pb-based ferroelectric in electronic device application.

Lead-free ferroelectric materials, such as Bi_0.5_Na_0.5_TiO_3_, are candidates for replacing lead-based materials because of their improved properties^19^. The strong polarization in Bi_0.5_Na_0.5_TiO_3_ materials could be due to the lone pair effect of Bi^3+^ compared to that of Pb^2+^ in the perovskite structure^20,21^. Several materials possessing a perovskite structure exist as a well solid solution in Bi_0.5_Na_0.5_TiO_3_ materials, thereby exhibiting enhanced performance. BiAlO_3_-modified Bi_0.5_Na_0.5_TiO_3_ materials as a solid solution strongly enhance the electric field-induced strain^22^. Kang *et al*. reported that a BaTiO_3_ solid solution in Bi_0.5_Na_0.5_TiO_3_ materials has the potential for energy harvesting^23^. Furthermore, Lin *et al*. reported that a Bi_0.5_Li_0.5_TiO_3_ solid solution in Bi_0.5_Na_0.5_TiO_3_ decreased the sintering temperature and enhanced the piezoelectric coefficient up to 121 pC N^−1^ ^24^. BiAlO_3_ materials are generally fabricated under extreme conditions. Although the properties of Bi_0.5_Li_0.5_TiO_3_ materials are not well known, they exist as a solid solution with enhanced performance properties relative to their host materials^25,26^. Therefore, modified Bi_0.5_Na_0.5_TiO_3_ materials exhibit excellent properties highlighting their potential in fabricating electronic devices.

The magnetic properties of Bi_0.5_Na_0.5_TiO_3_ materials have been investigated recently. The replacement of Ti cations with Co and Fe cations at the octahedral site of Bi_0.5_Na_0.5_TiO_3_ materials resulted in ferromagnetic ordering at room temperature^27,28^. Ferromagnetism in Fe-doped Bi_0.5_Na_0.5_TiO_3_ materials is an intrinsic phenomenon, whereas ferromagnetism in Co-doped Bi_0.5_Na_0.5_TiO_3_ materials is due to the presence of magnetic Co clusters^27,28^. Thanh *et al*. reported that Mn- and Cr-doped Bi_0.5_Na_0.5_TiO_3_ materials possibly influence room-temperature ferromagnetism^29,30^. On the other hand, the room-temperature ferromagnetic properties in Mn-doped Bi_0.5_Na_0.5_TiO_3_ materials result from an interaction of the Mn cation through O vacancies, whereas those in Cr-doped Bi_0.5_Na_0.5_TiO_3_ materials were mostly related to self-defects and enhancement via O vacancies^29,30^. Zhang *et al*. predicted that a Na or Ti vacancy, rather than a Bi or O vacancy, could induce magnetism^31^. Ju *et al*. predicted that substituting a Ti atom with a transition metal produces magnetic moments because of the spin polarization of 3*d* electrons in a transition metal^32^. Therefore, room-temperature ferromagnetism in lead-free ferroelectric Bi_0.5_Na_0.5_TiO_3_ materials could be the result of the introduction of a single transition metal into their host lattice. On the other hand, the limitation in the number of transition-metal-doped lead-free Bi_0.5_Na_0.5_TiO_3_ materials has resulted in poor ferromagnetism performance, such as low magnetization at room temperature (approximately several memu/g at room temperature), suggesting a real application in electronic devices. Given the desirable solid solution with *AB*O_3_-type materials, the magnetization of Bi_0.5_Na_0.5_TiO_3_ materials is enhanced strongly^33–36^. Hue *et al*. reported that the presence of an ilmenite-type material, such as MnTiO_3_ or NiTiO_3_ solid solution, in the host Bi_0.5_Na_0.5_TiO_3_ material could enhance magnetization^33,34^. This enhancement was also achieved when perovskite-type MgFeO_3_-_δ_ and SrFeO_3_-_δ_ solid solutions were added to the host Bi_0.5_Na_0.5_TiO_3_ materials^35,36^.

Among members of the alkaline-earth iron-based perovskite *Ae*FeO_3-δ_ family (*Ae* = Ba, Ca, Sr, and Mg), CaFeO_3-δ_ is interesting because the O deficiency could control the structural and magnetic properties^35–41^. Ceretti *et al*. reported that CaFeO_2.5_ exhibits a brown millerite structure^37^. Tassel *et al*. revealed that CaFeO_2_ has a layered structure, and Takeda *et al*. reported that CaFeO_3_ exhibits a perovskite structure^38,39^. The latter has antiferromagnetic ordering, with a Neel temperature of 120 K^39^. In addition, CaFeO_2.5_ exhibits an antiferromagnetic *G*-type structure, with a Neel temperature range of 700–725 K^36,40^. CaFeO_2_ also shows *G*-type antiferromagnetic order, with a Neel temperature of 420 K^41^. Recently, MgFeO_3_-_δ_ and SrFeO_3_-_δ_ were reported to forma well solid solution in host Bi_0.5_Na_0.5_TiO_3_ materials, resulting in greater magnetization than those in single-transition-metal-doped Bi_0.5_Na_0.5_TiO_3_ materials^35,36^. The enhancement of magnetization in alkaline-earth iron-based perovskite-modified Bi_0.5_Na_0.5_TiO_3_ materials possibly originated from the co-modification of both *A*- and *B*-sites with alkaline-earth and transition metals. Therefore, CaFeO_3-δ_-modified Bi_0.5_Na_0.5_TiO_3_ materials may also exhibit high magnetization through the diffusion of Ca and Fe cations in the host lattice and incorporation during the formation of the solid solution. In this study, a new (1-*x*)Bi_0.5_Na_0.5_TiO_3_-*x*CaFeO_3-δ_ solid solution compound was prepared using sol–gel technique. Bi_0.5_Na_0.5_TiO_3_ materials with CaFeO_3-δ_ maintained their original rhombohedral structure. Their distorted structure was attributed to the random distribution of Ca and Fe cations. Bi_0.5_Na_0.5_TiO_3_ samples with CaFeO_3-δ_ exhibited stronger room-temperature ferromagnetism than pure Bi_0.5_Na_0.5_TiO_3_ materials or single-transition-metal-doped Bi_0.5_Na_0.5_TiO_3_. These intrinsic and tunable ferromagnetism properties of undoped and doped Bi_0.5_Na_0.5_TiO_3_ were investigated further through density functional theory (DFT) calculations.

## Results

### Room temperature structure

**Figure 1:**
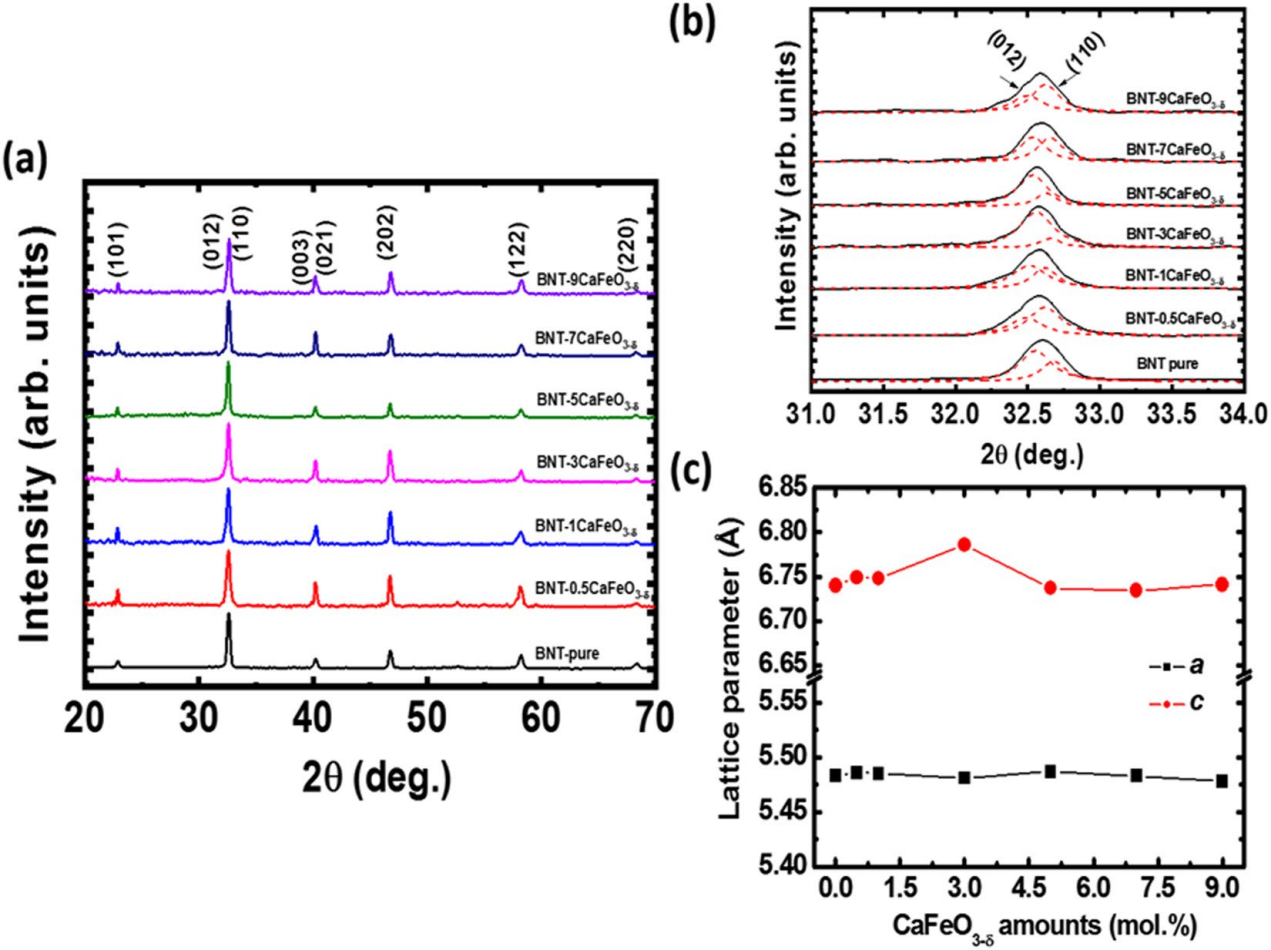
(**a**) XRD spectra (**b**) its deconvolution ranged from 31.0° to 34.0° and (**c**) lattice constants of pure and CaFeO_3–δ_-modified Bi_0.5_Na_0.5_TiO_3_ materials as solid solution at 0.5, 1, 3, 5, 7 and 9 mol.%.

The X-ray diffraction (XRD) patterns of CaFeO_3-δ_-modified Bi_0.5_Na_0.5_TiO_3_ with a CaFeO_3-δ_ concentration of up to 9 mol.% showed that CaFeO_3-δ_ was well dissolved in the host Bi_0.5_Na_0.5_TiO_3_ crystal. Figure 1(a) shows the XRD patterns of CaFeO_3-*δ*_-modified Bi_0.5_Na_0.5_TiO_3_ samples with various CaFeO_3-*δ*_ concentrations. All relative peak positions and intensities were indexed to rhombohedral symmetry, indicating that the crystalline structure of CaFeO_3-*δ*_-modified Bi_0.5_Na_0.5_TiO_3_ samples adopted the crystal structure of the host Bi_0.5_Na_0.5_TiO_3_ materials. Furthermore, CaFeO_3-*δ*_ existed in form of a solid solution in Bi_0.5_Na_0.5_TiO_3_ through the diffusion of Ca and Fe cations and incorporation in the host lattice. The impurity phase was not determined by XRD owing to its resolution limit. The Ca and Fe cations modified the lattice parameter of Bi_0.5_Na_0.5_TiO_3_ materials, as shown in Fig. 1(b), where the diffraction angles 2*θ* increased by 31.0°–34.0°. A broad peak position was obtained because of the overlap of two diffraction peaks, which complicated their comparison. Each XRD peak was distinguished using Lorentz fitting, as shown by the red dotted line in Fig. 1(b). Furthermore, the lattice parameters *a* and *c* of the pure Bi_0.5_Na_0.5_TiO_3_ and the CaFeO_3–δ_-modified Bi_0.5_Na_0.5_TiO_3_ according to CaFeO_3–δ_ addition amounts is shown in Fig. 1(c). The results show that distorted lattice parameters of Bi_0.5_Na_0.5_TiO_3_ compound is not linear as function of CaFeO_3–δ_ amounts, that has complexed distortion in lattice parameters. This could be attributed to cation radius difference between Ca and Fe in additives and Bi, Na and Ti of host material incorporating randomly with host lattice. Based on Shannon’s report, the radii of Ca^2+^, Bi^3+^, and Na^+^ cations in coordination number 12 are 1.34, 1.17, and 1.39 Å, respectively, whereas those of Fe^3+/2+^ and Ti^4+^ cations in a coordination number 6 are 0.645 Å/0.780 Å and 0.605 Å, respectively^42^. Therefore, the average radius of the *A*-site of (Bi_0.5_Na_0.5_)^2+^ is 1.28 Å, which is smaller than that of Ca^2+^ (1.34 Å). On the other hand, the lattice parameters expanded when the Ca^2+^ cations substituted Bi^3+^ cations in the host lattice, where the lattice parameters were reduced when Ca^2+^ cations replaced the Na^+^ cations. Moreover, to maintain a balanced charge, Ca^2+^ acted as an acceptor for replacing Bi^3+^ cations, resulting in the formation of O vacancies, and Ca^2+^ cations acted as a donor for incorporating Na^+^ cations, thereby producing Na-vacancies. Similarly, the radii of Fe^2+/3+^ cations are larger than that of Ti^4+^. Therefore, the distorted crystal structure of Bi_0.5_Na_0.5_TiO_3_ could be attributed to the replacement of Ti^4+^ cations with large Fe^2+/3+^ cations. Based on the Hume–Rothery rules, Ca^2+^ cations enter at the substituted *A*-site of Bi_0.5_Na_0.5_TiO_3_ materials because of the 4.7% difference between the radius of Ca^2+^ and the average radius of (Bi_0.5_Na_0.5_)^2+^^43–45^. The differences between the radii of Fe^3+^ and Fe^2+^ cations and those of Ti^4+^ cations substituted at the *B*-site of Bi_0.5_Na_0.5_TiO_3_ materials are 6.6% and 28.9%, respectively, which, according to the Hume–Rothery rules, is too large to allow replacement because of the increased lattice energy. The lattice energy can be reduced if the difference in the sizes between the O vacancies and O anion is consistent. O vacancies were formed because of the unbalanced charges between Fe^3+/2+^ and Ti^4+^. Chatzichristodoulou *et al*. reported that the effective radius of O vacancies (1.31Å) is smaller than that of the O anion ion (1.4Å), resulting in a decrease lattice constants^46^. The flaccidity of the size of O vacancies on the lattice parameters has a more significant influence than that of dopants in perovskite Bi_0.5_Na_0.5_TiO_3_ or BaTiO_3_ materials^30,47^. Therefore, CaFeO_3-*δ*_ materials exists as a well solid solution in the Bi_0.5_Na_0.5_TiO_3_ structure and distort the crystal structure of the latter.

**Figure 2:**
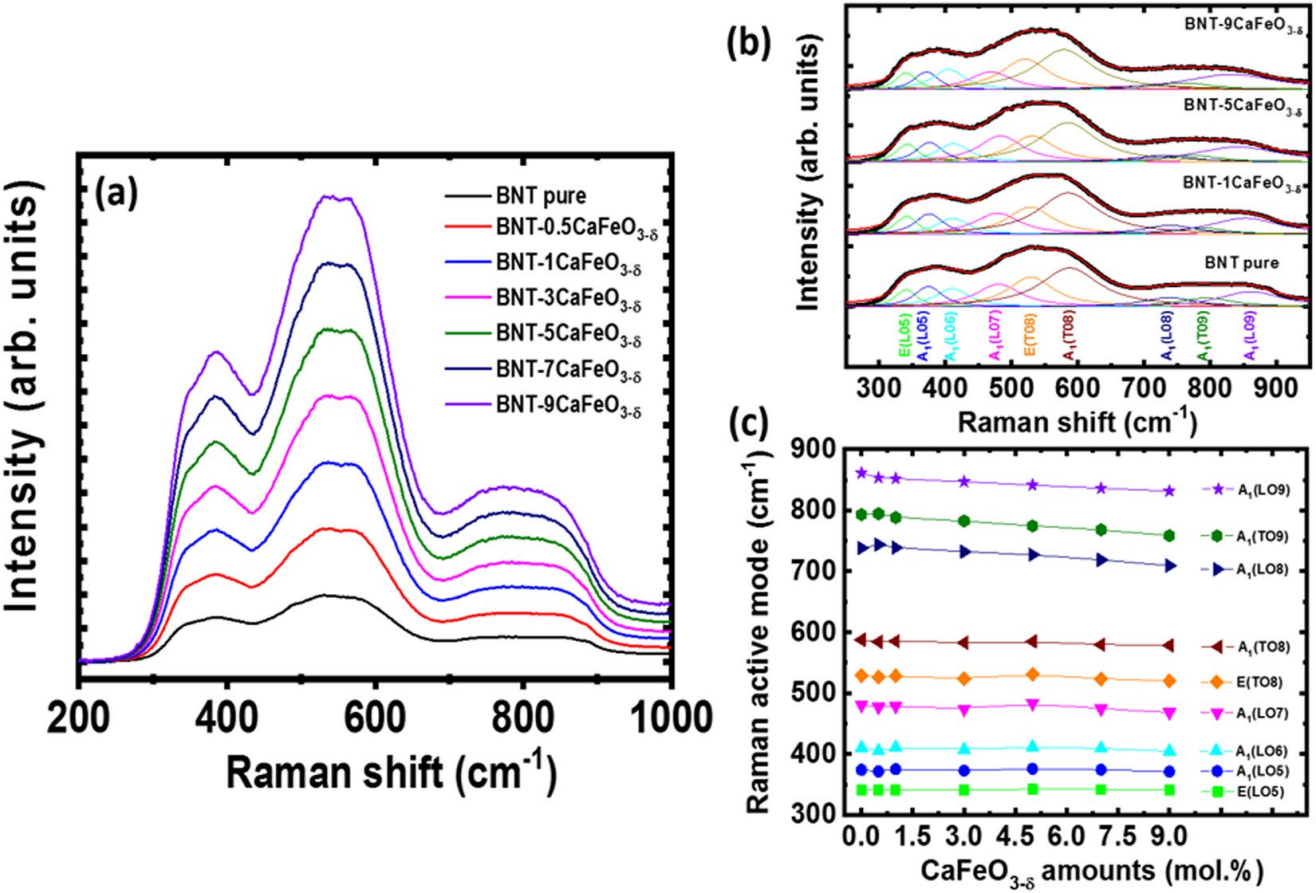
(**a**) Raman spectra of pure Bi_0.5_Na_0.5_TiO_3_ and CaFeO_3-*δ*_-modified Bi_0.5_Na_0.5_TiO_3_ samples as solid solutions with various concentrations, (**b**) deconvolutions of the Raman spectra of pure Bi_0.5_Na_0.5_TiO_3_ and CaFeO_3-*δ*_ solid solutions in Bi_0.5_Na_0.5_TiO_3_ with 1, 5, and 9 mol% CaFeO_3-*δ*_, and (**c**) Raman actives modes of Bi_0.5_Na_0.5_TiO_3_ dependence on the CaFeO_3-*δ*_ amount as solid solution.

The solute solution of CaFeO_3-δ_ into host Bi_0.5_Na_0.5_TiO_3_ materials was further confirmed using Raman scattering studies. Figure 2(a) shows the Raman spectra of Bi_0.5_Na_0.5_TiO_3_ and CaFeO_3-δ_-modified Bi_0.5_Na_0.5_TiO_3_ materials within range of 200 and 1000 cm^−1^. The Raman spectra of the undoped Bi_0.5_Na_0.5_TiO_3_ and CaFeO_3-δ_-modified Bi_0.5_Na_0.5_TiO_3_ materials exhibited similar shapes. Therefore, the vibration modes of CaFeO_3-δ_-modified Bi_0.5_Na_0.5_TiO_3_ materials were similar to those of the undoped Bi_0.5_Na_0.5_TiO_3_ materials. This conforms to the XRD patterns, suggesting that the CaFeO_3-δ_-modified Bi_0.5_Na_0.5_TiO_3_ materials maintained their original crystal structural of host Bi_0.5_Na_0.5_TiO_3_ compounds. On the other hand, the Raman scattering spectra of pure and CaFeO_3-δ_-modified Bi_0.5_Na_0.5_TiO_3_ were separated approximately into three overlapping active bands: 300–450, 450–700, and 700–1000 cm^−1^. The overlap in the Raman scattering modes may originate from the random distribution of Na and Bi cations at the *A*-site in the perovskite structure^48^. In addition, experimental and theoretical investigations both predicted that the lowest frequency modes within 246–401 cm^−1^ are related to the TiO_6_ vibration, whereas the highest frequency modes within 413–826 cm^−1^ are due to the vibration of O atoms^48^. Chen *et al*. assigned the Raman scattering in the range of 200–400 cm^−1^ to Ti-O vibration, whereas the Raman scattering in the range of 450–700 cm^−1^ is related to the TiO_6_ octahedral vibration^49^. Hence, distinguishing each mode and comparing the roles of Ca and Fe cations in the lattice vibration are difficult. Despite this, an attempt was made to distinguish the Raman modes for pure Bi_0.5_Na_0.5_TiO_3_ and CaFeO_3-δ_-modified Bi_0.5_Na_0.5_TiO_3_ materials using a Lorentz fit within the range of 250–950 cm^−1^. The Raman active modes within the said wave-number range were obtained with a correction of fitting over 0.99. Figure 2(b) shows the Raman modes for pure Bi_0.5_Na_0.5_TiO_3_ and CaFeO_3-*δ*_-modified Bi_0.5_Na_0.5_TiO_3_ with 1, 5, and 9 mol.%. Each active mode was well indexed based on the calculation and experimental results for the active Raman modes in Bi_0.5_Na_0.5_TiO_3_ materials^48^. The dependent of Raman active peak modes on the CaFeO_3-*δ*_ amounts solid solution into host Bi_0.5_Na_0.5_TiO_3_ materials was shown in Fig. 2(c). The results clearly show that the Raman peaks shifted to lower frequency as increase of CaFeO_3-*δ*_ concentration. However, the shift of Raman peak frequencies was not decreased linearly to the CaFeO_3-*δ*_ concentration, but has complex function. Normally, the increase in the ionic radii results in a distortion of the structure, leading to a high frequency shift, whereas the increase in the mass results in a low-frequency shift^49^. The XRD peaks for Bi_0.5_Na_0.5_TiO_3_ materials shifted to low diffraction angles. Therefore, the Raman scattering modes were expected to shift to a high frequency. On the other hand, the mass values of the Ca and Fe cations were larger than those of the average *A*-site (Bi, Na) and Ti cations, possibly leading to a low-frequency shift. Thus, the low-frequency shifts in Raman scattering modes were related to the (Ti,Fe)O_6_ vibration modes. In addition, compared to the average of (Bi, Na), the mass values of Bi and Na cations (*m*_Bi_ = 208.98 and *m*_Na_ = 22.99) at the *A*-site of 164.99 were larger than that of calcium (*m*_Ca_ = 40.08), whereas the mass of the Ti cation (*m*_Ti_ = 47.86) was smaller than that of the Fe cation (*m*_Fe_ = 55.85). The shift of the Raman vibration modes confirmed the random substitution of Ca and Fe cations into the host lattice of Bi_0.5_Na_0.5_TiO_3_ materials; this substitution occurred because of the difference in the mass values of Ca and Fe cations compared to those of (Bi, Na) and Ti, respectively, and the distorted structure of the samples. In other words, the shifted Raman scattering modes confirmed the incorporation of Ca and Fe into the host lattice of the Bi_0.5_Na_0.5_TiO_3_ materials.

### Optical properties

**Figure 3:**
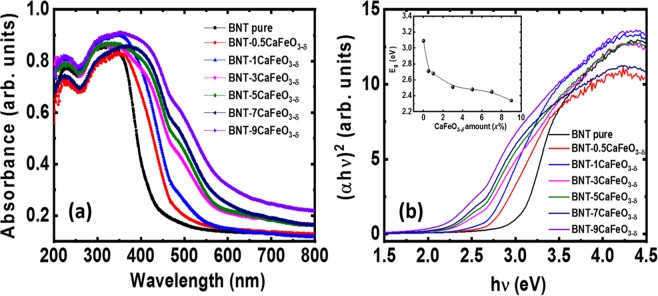
(**a**) UV-Vis absorption spectra of pure Bi_0.5_Na_0.5_TiO_3_ and (1-*x*)Bi_0.5_Na_0.5_TiO_3_ + *x* CaFeO_3-*δ*_ samples as the function of CaFeO_3-*δ*_ solid solution, and (**b**) the (*α*h*ν*)^2^ proposal with photon energy (h*ν*) of pure Bi_0.5_Na_0.5_TiO_3_ as the function of the CaFeO_3-*δ*_concentration solid solution. Inset of Fig. 3(b) shows the optical band gap of the Bi_0.5_Na_0.5_TiO_3_ samples as a function of the CaFeO_3-*δ*_ concentrations.

The solute solution of CaFeO_3-δ_ into the host Bi_0.5_Na_0.5_TiO_3_ materials results in a decrease in the optical band gap. Figure 3(a) presents the optical absorbance spectra of undoped Bi_0.5_Na_0.5_TiO_3_ and CaFeO_3-δ_-modified Bi_0.5_Na_0.5_TiO_3_ materials at various concentrations at room temperature. The pure Bi_0.5_Na_0.5_TiO_3_ samples exhibited a single absorbance edge, which is consistent with the recently reported optical properties of Bi_0.5_Na_0.5_TiO_3_ materials^29,30,50,51^. The addition of CaFeO_3-δ_ to Bi_0.5_Na_0.5_TiO_3_ caused the absorbance edge to shift to high wavelengths, indicating that the electronic band structures had been modified. Furthermore, the absorbance spectra of the CaFeO_3-δ_-modified Bi_0.5_Na_0.5_TiO_3_ samples showed absorbance peaks at approximately 485 nm, indicating the local states of the Fe cations. This result is consistent with the recent observation of the absorbance spectra of Fe cation impurities in Bi-based ferroelectric materials, such as Bi_0.5_K_0.5_TiO_3_ and Bi_0.5_Na_0.5_TiO_3_ materials^35,36,52^. In addition, CaFeO_3-δ_-modified Bi_0.5_Na_0.5_TiO_3_ materials exhibited smooth absorbance edges with slight tails. The appearance of tails in CaFeO_3-δ_-modified Bi_0.5_Na_0.5_TiO_3_ materials could be related to an intrinsic defects or surface effects^51^. The optical band gap (*E*_g_) was estimated using the Wood and Tauc method^53^. In this approach, the *E*_g_ values are associated with the absorbance and photon energy, as shown by the following relation: (*α*h*ν*)~(h*ν*-*E*_g_)^*n*^, where *α, h*, and *ν* are the absorbance coefficient, Planck constant, and frequency, respectively; *n* is a constant related to different types of electronic transition (*n* = 1/2, 2, 3/2, or 3 for directly allowed, indirectly allowed, directly forbidden, or indirectly forbidden transition, respectively)^53^. Thus, *E*_g_ can be evaluated by extrapolating the linear portion of the curve or tail from the intercept of (*α*h*ν*)^1/*n*^ versus the photon energy *hν*. A calculation of the electronic band structure showed that Bi_0.5_Na_0.5_TiO_3_ has a direct band gap of 2.1 eV, and the optical spectra of Bi_0.5_Na_0.5_TiO_3_ were determined mainly by the contributions from the O 2*p* valence bands to the Ti 3*d* and Bi 6*p* conduction bands in the low-energy region^54^. Therefore, *n* = 1/2 for direct transition was used, as shown in Fig. 3(b). Pure Bi_0.5_Na_0.5_TiO_3_ materials exhibited an *E*_g_ of approximately 3.11 eV, whereas CaFeO_3-δ_-modified Bi_0.5_Na_0.5_TiO_3_ materials showed lower *E*_g_ value (2.34 eV for 9 mol.% CaFeO_3-δ_ solid solution in Bi_0.5_Na_0.5_TiO_3_). The inset of Fig. 3(b) shows the dependence of *E*_g_ on the amount of CaFeO_3-δ_-modified Bi_0.5_Na_0.5_TiO_3_ materials. The decrease in the optical band gap in CaFeO_3-δ_-modified Bi_0.5_Na_0.5_TiO_3_ materials possibly originated from the random distribution of Ca and Fe cations into the host lattice of the Bi_0.5_Na_0.5_TiO_3_ materials. The replacement of a Ti cation with a transition metal, such as Mn and Cr, in the Bi_0.5_Na_0.5_TiO_3_ materials resulted in a decrease in the optical band gap because the impurities of these transition metal cations formed new local states in the middle of the electronic band structure^29,30^. In addition, the appearance of O vacancies located near the conduction band also affected the optical band gap of the Bi_0.5_Na_0.5_TiO_3_ materials^29,30^. Note that O vacancies were generated due to the unbalanced charges between Fe^2+/3+^ that substituted for Ti^4+^ at the octahedral site and Ca^2+^ cations that replaced Bi^3+^. In addition, the O vacancies located in the crystal structure promoted the valence transition of Ti^4+^ to Ti^3+^^55^. The appearance of new state Na + -vacancies or Ti^3+^-defect also contributed to the decrease in the optical band gap. Recently, *A*-site modified Bi_0.5_Na_0.5_TiO_3_-based material showed the decline of the optical band gap because of the changes in the bond type between the hybridization of *A*-O in the *AB*O_3_ perovskite structures^56,57^. Thus, the random substitution of Ca and Fe ions into the host Bi_0.5_Na_0.5_TiO_3_ changed the electronic band structure and decreased the optical band gap.

**Figure 4:**
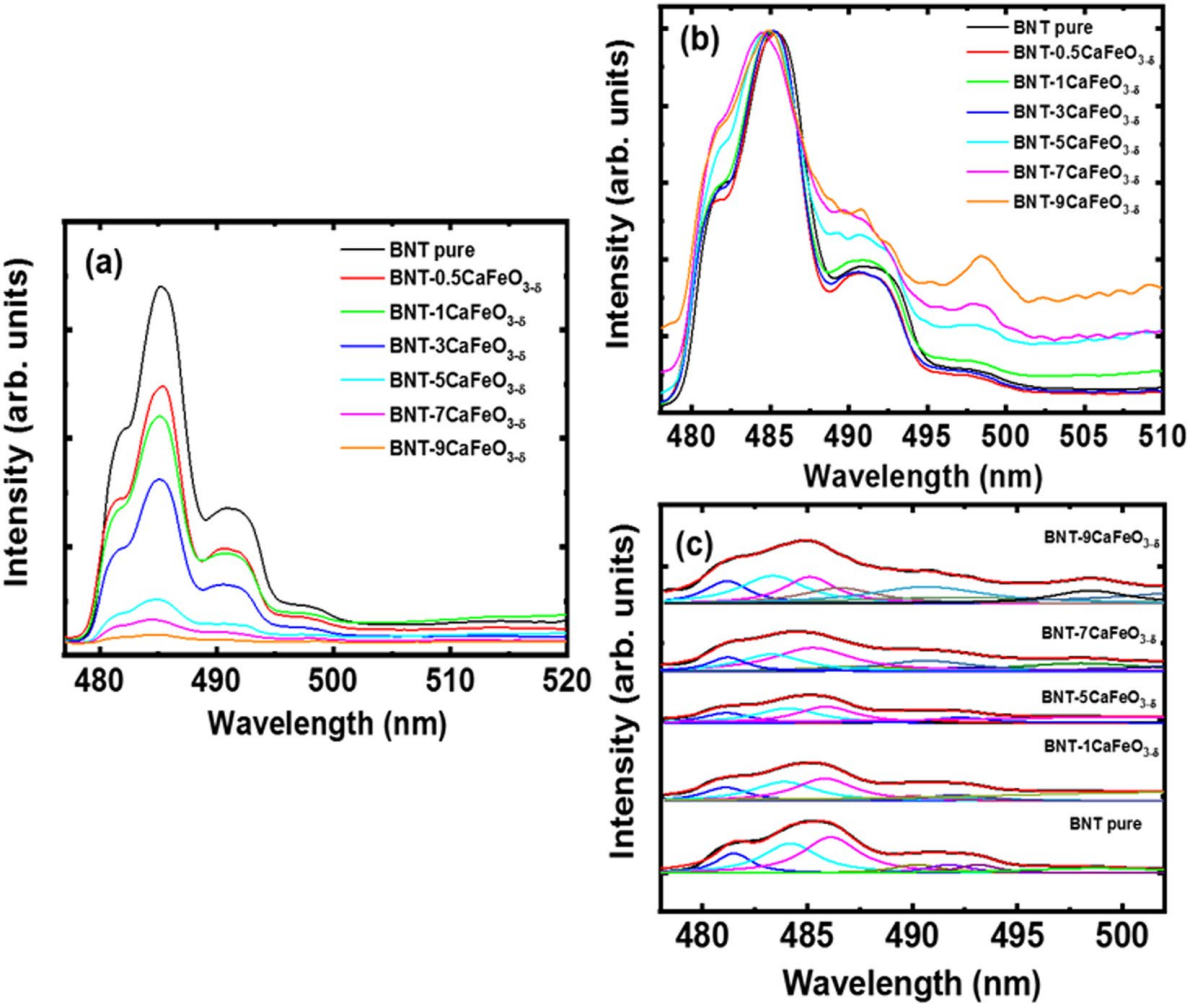
(**a**) Real, (**b**) normalized PL spectra, and (**c**) its deconvolutions in pure Bi_0.5_Na_0.5_TiO_3_ samples, and Bi_0.5_Na_0.5_TiO_3_ solid solution with various CaFeO_3-*δ*_ concentrations.

The CaFeO_3-δ_-modified Bi_0.5_Na_0.5_TiO_3_ materials as a solid solution suppressed the photoluminescence (PL) of host materials. Figure 4(a) shows the room-temperature PL emission spectra of the Bi_0.5_Na_0.5_TiO_3_ samples. The spectra clearly showed a broad blue emission band within 476–505 nm. The PL intensity of Bi_0.5_Na_0.5_TiO_3_ materials decreased with increasing amount of CaFeO_3-δ_ solid solution in the Bi_0.5_Na_0.5_TiO_3_ materials. The strong PL peak positions of pure Bi_0.5_Na_0.5_TiO_3_ materials and CaFeO_3-δ_-modified Bi_0.5_Na_0.5_TiO_3_ materials were compared via subtraction to the unit, as shown in Fig. 4(b). The peak showed a blue shift as the CaFeO_3-δ_ concentration was increased. The broad band emission peak of pure Bi_0.5_Na_0.5_TiO_3_ materials and CaFeO_3-δ_-modified Bi_0.5_Na_0.5_TiO_3_ materials with 1, 5, 7, and 9 mol.% CaFeO_3-δ_ were deconvoluted by a Lorentzian fit with the roughest square of more than 0.99, as shown in Fig. 4(c). The PL of ferroelectric materials is not generally dominated by a band-to-band transition, considering the difficulty in combining electron–hole pairs due to the separation of the nature polarization domain in the materials. On the other hand, the surface states are the dominant cause of luminescence in perovskites. Numerous unsaturated atoms exist on the surface of the perovskites, forming localized levels within the forbidden gaps of the materials. Lin *et al*. reported that a self-trapped excitation possibly originated from the PL of the Bi_0.5_Na_0.5_TiO_3_ materials, whereas the distortion of the TiO_6_ octahedra due to surface stress resulted in a blue shift in the emission peak^58^. Bac *et al*. also reported the disordered coupling to a tilt of the TiO_6_-TiO_6_ adjacent octahedral that resulted in structural distortion and generation of localized electronic levels above the valence band; these phenomena are mainly responsible for the PL emission of Bi_0.5_K_0.5_TiO_3_ materials^51^. Interestingly, the addition of CaFeO_3-δ_ reduced the PL emission intensity of the Bi_0.5_Na_0.5_TiO_3_ materials (Fig. 4[a]), possibly by trapping electrons generated from absorbance photon energy that prevented electron–hole recombination to generate photons through the defects.

### Magnetic properties

**Figure 5:**
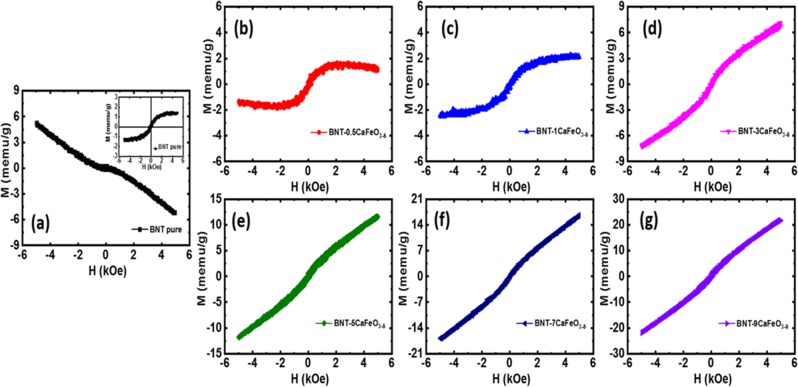
M–H curves of (1-*x*)Bi_0.5_Na_0.5_TiO_3_ + *x*CaFeO_3-*δ*_ concentration solid solution with various amounts.

The complex magnetic properties at room temperature of Bi_0.5_Na_0.5_TiO_3_ materials were measured as a function of the CaFeO_3-δ_ solute solution. Figure 5(a–g) show the magnetic hysteresis loops (M–H) of pure Bi_0.5_Na_0.5_TiO_3_ materials and CaFeO_3-δ_-modified Bi_0.5_Na_0.5_TiO_3_ materials with various amounts of CaFeO_3-δ_ (0.5, 1, 3, 5, 7, and 9 mol.%) at room temperature. The pure Bi_0.5_Na_0.5_TiO_3_ materials exhibited an anti-*S*-shape M–H curve, which was attributed to the compensation of the diamagnetism of the empty 3*d* orbital of Ti and weak ferromagnetism of intrinsic defects or surface defects. The critical S-shape in the M–H curve of the ferromagnetic thin films were obtained in pure Bi_0.5_Na_0.5_TiO_3_ materials after subtracting diamagnetism components, as shown in the inset of Fig. 5(a). The saturation of magnetization was approximately 1.5 memu g^−1^, which is similar to the results of recent reports^30,59^. The slightly addition of CaFeO_3-δ_ amounts to the host Bi_0.5_Na_0.5_TiO_3_ materials give rise to reduction of diamagnetic components, as shown in Fig. 5(b). The M–H curve was saturated under the applied external magnetic field for 1 mol.% CaFeO_3-δ_-modified Bi_0.5_Na_0.5_TiO_3_ materials as a solid solution, as shown in Fig. 5(c), further confirming the ferromagnetic state ordering at room temperature. On the other hand, the unsaturation magnetization under the applied magnetic field was obtained with the further addition of CaFeO_3-δ_ in Bi_0.5_Na_0.5_TiO_3_ materials as solid solution, as shown in Fig. 5(d–g). The maximum magnetization was approximately 21.6 memu g^−1^ for 9 mol.% CaFeO_3-δ_ solid solution in Bi_0.5_Na_0.5_TiO_3_ materials. These results suggest strong enhancement of the magnetization of CaFeO_3-δ_-modified Bi_0.5_Na_0.5_TiO_3_ materials, which is greater than that of pure Bi_0.5_Na_0.5_TiO_3_ (~1.5 memu g^−1^) or transition-metal-doped Bi_0.5_Na_0.5_TiO_3_ materials (~1.5–2 memu g^−1^ for Cr-doped Bi_0.5_Na_0.5_TiO_3_, ~3 memu g^−1^ for Co-doped Bi_0.5_Na_0.5_TiO_3_, ~9 memu g^−1^ for Mn-doped Bi_0.5_Na_0.5_TiO_3_, and ~11 memu g^−1^ for Fe-doped Bi_0.5_Na_0.5_TiO_3_)^27–30,59^. CaFeO_3_, CaFeO_2.5_, and CaFeO_2_ compounds have antiferromagnetic ordering, with Neel temperatures of 120, 700–725, and 420 K, respectively^36,40,41^. In the formation of a solid solution, however, the CaFeO_3-δ_-modified Bi_0.5_Na_0.5_TiO_3_ samples exhibited greater room-temperature ferromagnetism than single-transition-metal-doped Bi_0.5_Na_0.5_TiO_3_ materials. Therefore, modification of the *A*-site in perovskite, together with the presence of a transition metal at the *B*-site in lead-free ferroelectric materials, is important for the current integration and development of magnetism for ferroelectric materials. The possible mechanisms of room-temperature ferromagnetic ordering in transition-metal-doped Bi_0.5_Na_0.5_TiO_3_ materials were as follows: (*i*) interaction of a magnetic cation through O vacancies, such as the *F*-center mechanism^28,29^, (*ii*) enhanced magnetism of O vacancies^30^, and (*iii*) magnetism of clusters embedded in the host materials^27^. Unlike that of single-transition-doped Bi_0.5_Na_0.5_TiO_3_ materials, the *A*-site of Bi_0.5_Na_0.5_TiO_3_ materials was modified by Ca, causing complications, such as Na and O vacancies (□). Both defects possibly induced ferromagnetism. In addition, the risk of O vacancies promoted the valence transition from Ti^4+^ to Ti^3+^, thereby inducing ferromagnetism^55,60^. Moreover, the chemical strain due to the difference in the radii of Ca and Fe compared to that of the host lattice Bi_0.5_Na_0.5_TiO_3_ materials might have tuned the ferromagnetic ordering, such as the Fe^3+^-□-Fe^3+^ interaction or superinteraction of the magnetic polaron between [Fe^3+^-□-Fe^3+^] versus [Fe^3+^-□-Fe^3+^] etc. Of note, the tremendous interaction between polarons normally favored antiferromagnetic ordering, whereas the isolated Fe cations displayed paramagnetic ordering.

### Electronic band structure

**Figure 6:**
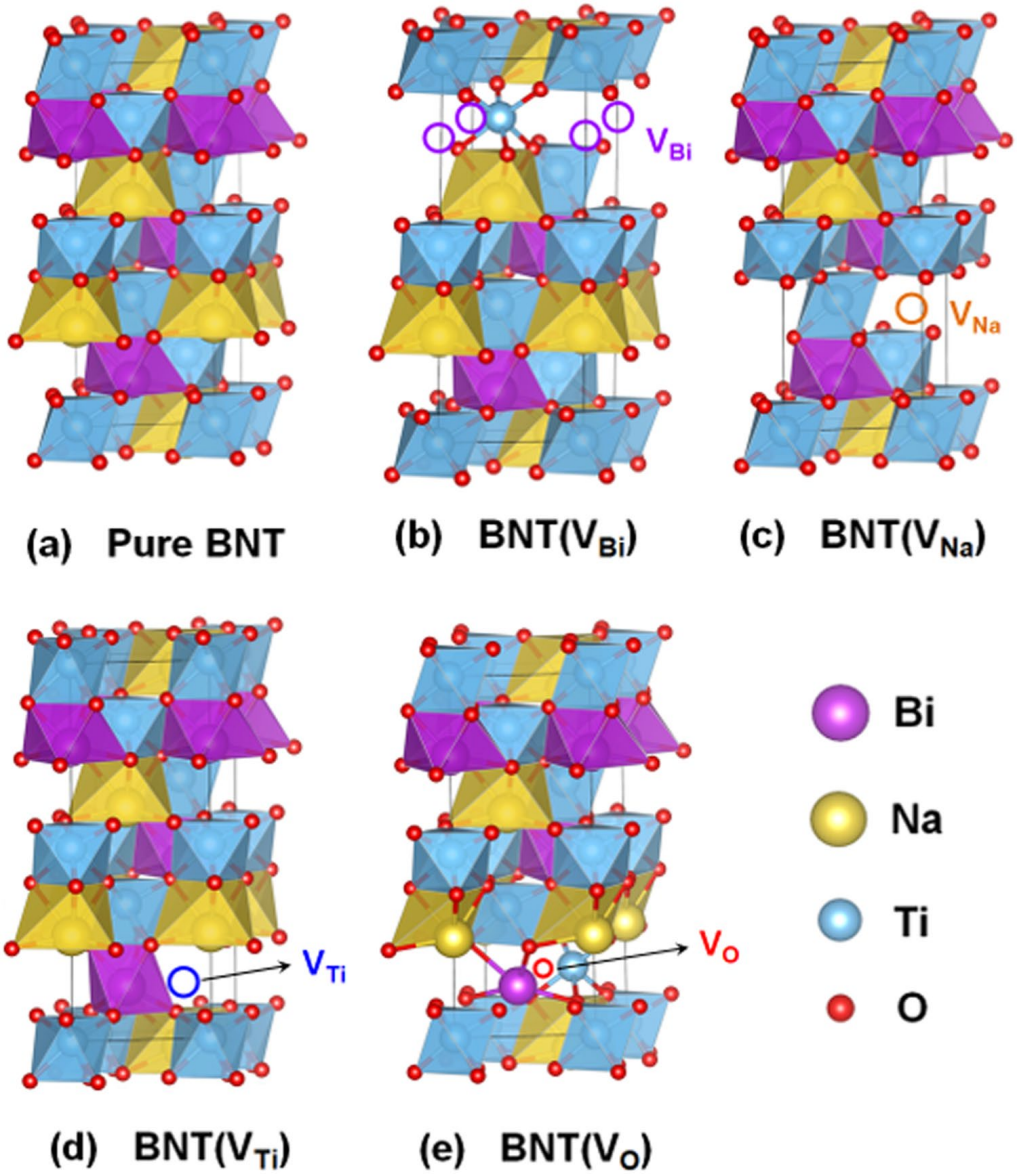
Optimized atomic structures of (**a**) BTN, (**b**) BNT(V_Bi_), (**c**) BNT(V_Na_), (**d**) BNT(V_Ti_), and (**e**) BNT(V_O_). The larger pink, yellow, and blue spheres denote the Bi, Na, and Ti atoms, respectively. The small red sphere indicates the O atom. The open-circle symbols represent the presence of various vacancies.

The intrinsic defects and random incorporation of Ca and Fe cations into the host lattice of Bi_0.5_Na_0.5_TiO_3_ materials give rise to the induced magnetism. To get insights on the weak ferromagnetism of Bi_0.5_Na_0.5_TiO_3_ with vacancy defects, we model all possible single atomic vacancies in the 6 and 24 formula unit (f.u.) cells of Bi_0.5_Na_0.5_TiO_3_. The corresponding vacancy concentrations are about 3.3 at.% (1 vacancy per 30-atom cell) and 0.83 at.% (1 vacancy per 120-atom cell), respectively. Figure 6(a–e) show the model geometries that are composed of 6 f.u. cells (for simplicity, 24 f.u. cell structures are omitted) of the rhombohedral structure for the pristine and defected Bi_0.5_Na_0.5_TiO_3_ with single Bi- [denoted as BNT(V_Bi_)], Na- [BNT(V_Na_)], Ti- [BNT(V_Ti_)], and O-site vacancy [BNT(V_O_)], respectively.

**Figure 7:**
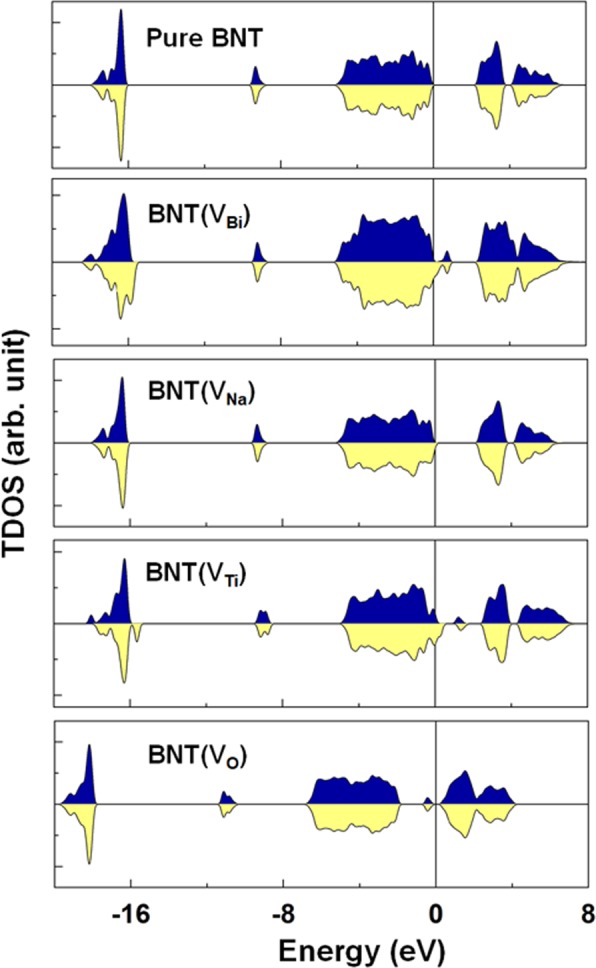
Top to bottom: Spin-decomposed total density of states (TDOS) of the BTN, BNT(V_Bi_), BNT(V_Na_), BNT(V_Ti_), and BNT(V_O_). The blue and yellow areas represent the majority-spin and minority-spin states, respectively. The Fermi level is set to zero energy.

The important role of intrinsic defects on the electronic band structure has been obtained using by DFT calculations. Figure 7 shows the spin-decomposed total density of states (TDOS) of the BNT, BNT(V_Bi_), BNT(V_Na_), BNT(V_Ti_), and BNT(V_O_) compounds. In the pure BNT, the majority- and minority-spin states were entirely degenerated, indicating the feature of a nonmagnetic ground state. The calculated band gap (~2.25 eV) of BNT is found to be somewhat smaller than the measured value of 3.08 eV, which is typical in DFT calculations for correlated oxide compounds^61^. The presence of an O vacancy shifts the band states downward and develops midgap states immediately below the Fermi level. This phenomenon is a reflection of the excess electrons (2 electrons per O vacancy) in the unit cell. Unlike the BNT(V_O_), the degeneracy of the spin sub-bands, particularly around the Fermi level, of the BNT does not persist anymore in the presence of the Bi, Na, and Ti vacancies. As shown in Table 1, the induced magnetism was approximately 0.13 (0.10) µ_B_ for the 6 (24) f.u. BNT(V_Bi_), 0.09 (0.0) µ_B_ for the 6 (24) f.u. BNT(V_Na_), and 0.30 (0.0) µ_B_ for the 6 (24) f.u. BNT(V_Ti_). Note that for the BNT(V_Na_) and BNT(V_Ti_) the induced magnetism showed the concentration dependence of the vacancy in the sample. On the other hand, the bandgap of the pure BNT remained relatively unchanged for the BNT(V_Na_), while it was reduced significantly for the BNT(V_Bi_) and BNT(V_Ti_). Our calculations indicate that a weak ferromagnetism of Bi_0.5_Na_0.5_TiO_3_ found in experiments can be the result of the formation of Bi or Na or Ti vacancies or, possibly, two or all of them during the sample growth.

**Table 1:** Magnetization per formula unit cell (μ_B_/f.u.) of Bi_0.5_Na_0.5_TiO_3_ with various vacancies for the 6 and 24 f.u. cells adopted in the DFT calculations.

Cell	BNT	BNT(V_Bi_)	BNT(V_Na_)	BNT(V_Ti_)	BNT(V_O_)
6-f.u	0.000	0.13	0.09	0.30	0.00
24-f.u	0.000	0.10	0.00	0.00	0.00

**Figure 8:**
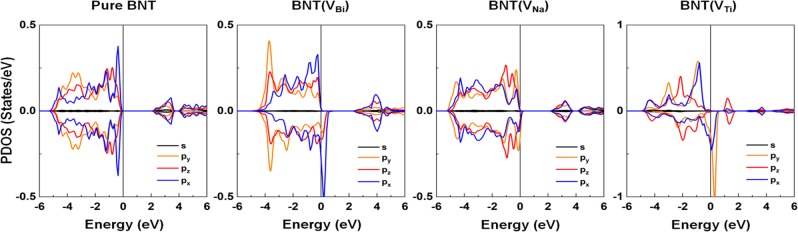
Left to right: The *s*- and *p*-orbital decomposed PDOS of the O atom for the BTN, BNT(V_Bi_), BNT(V_Na_), and BNT(V_Ti_). The black, orange, red, and blue lines represent the *s*, *p*_*y*_, *p*_*z*_, and *p*_*x*_ orbital states, respectively. The Fermi level is set to zero energy.

The further analyses with the atom and orbital projected DOS (PDOS) indicate that the vacancy induced magnetism mainly comes from the O atoms nearby the vacancy sites. We thus plot only the *s*- and *p*-orbital PDOS of the O atom for the BNT(V_Bi_), BNT(V_Na_), and BNT(V_Ti_) in Fig. 8. For comparison, the same for the pure BNT is also presented. For the BNT, the valence and conduction bands are characterized mainly by the O-2*p* and Ti-3*d* orbital states, respectively. Both Ti and O contributed to the filled midgap state in the BNT(V_O_), as shown in Fig. S3 in the supplemental data. As seen in Fig. 8, the O 2*p*_*x*,*y*_ orbital states play a main role for the induced magnetism of all systems, as the filled *p*_*x*,*y*_ orbital states in the minority-spin channel shift across the Fermi level into the unoccupied band region.

**Figure 9:**
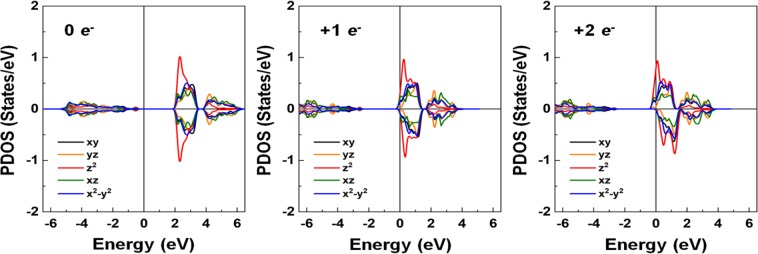
Left to right: The *d*-orbital decomposed PDOS of the Ti atom of the pristine BTN for zero, +1 *e*^*-*^, and +2 *e*^*-*^ injected charges. The black, orange, red, green, and blue lines represent the *d*_*xy*_, *d*_*yz*_, *d*_*z*_^2^, *d*_*xz*_, and *d*_*x*_^2^_–*y*_^2^ orbital states, respectively. The Fermi level is set to zero energy.

To imitate the presences of Ti^3+^ and Ti^2+^ valence states, we inject 1 and 2 *e*^*-*^ in the 6 f.u. cell of the pristine BNT and plot the *d*-orbital PDOS of the Ti atom in Fig. 9. This serves as a *n*-type doping, where the spin channel states split. The calculated magnetic moments are 0.083 and 0.32 µ_B_ per f.u. for 1 and 2 *e*^*-*^ doped BNT, respectively, which mainly resides at the Ti site. As expected, PDOS states move downward toward the Fermi level; the majority-spin states are partly occupied while the minority-spin states remain unoccupied.

**Figure 10:**
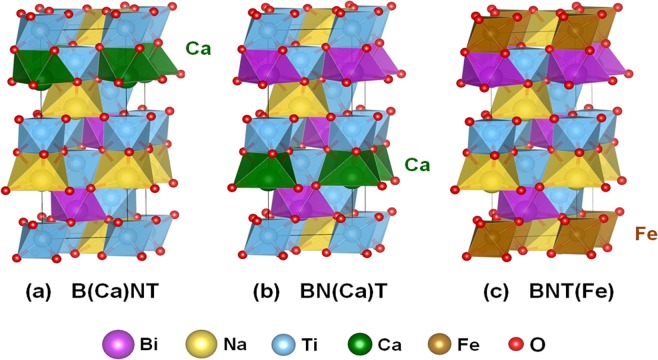
Optimized atomic structures of (**a**) B(Ca)TN, (**b**) BN(Ca)T, and (**c**) BNT(Fe). The larger pink, yellow, blue, green, and brown spheres denote the Bi, Na, Ti, Ca, and Fe atoms, respectively. The small red sphere indicates the O atom.

**Figure 11:**
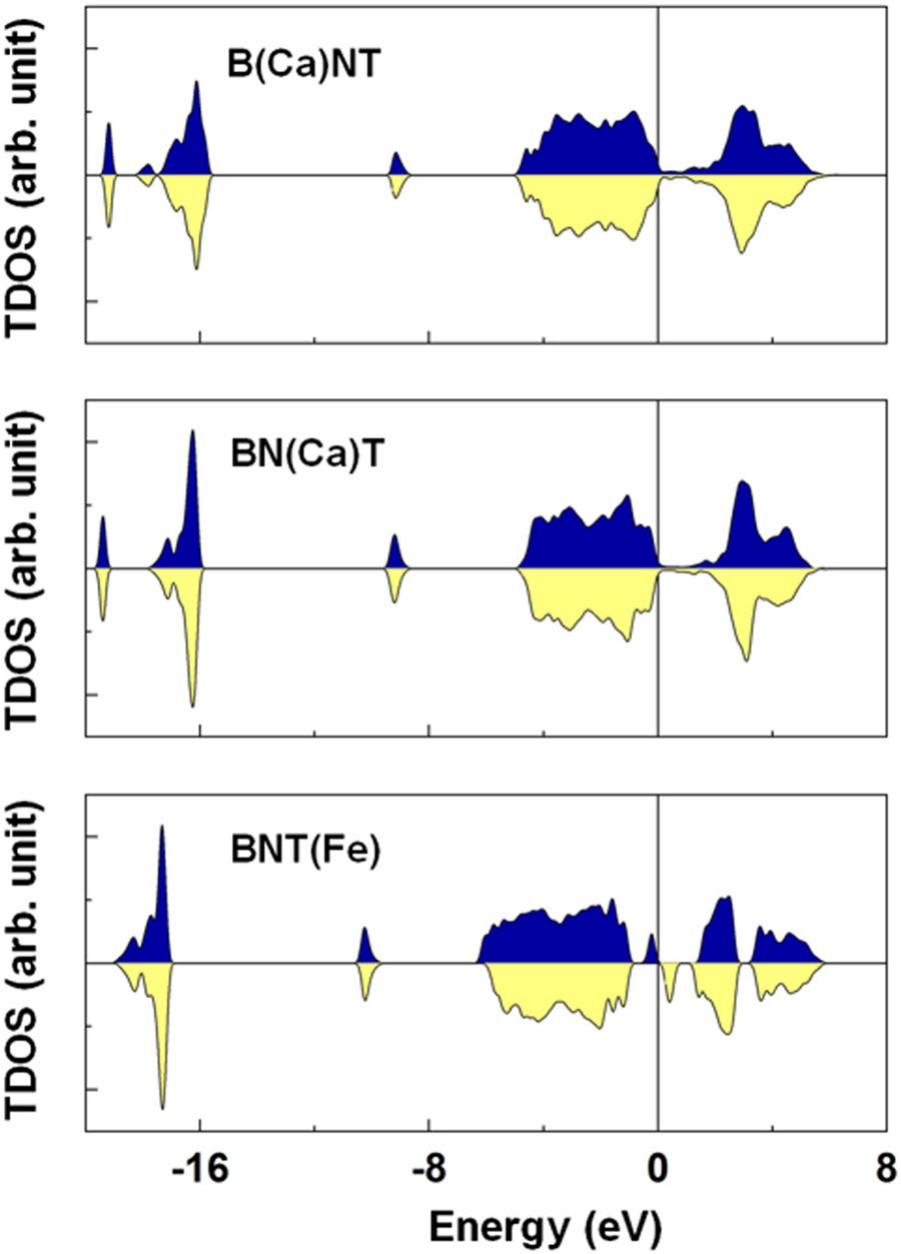
Top to bottom: Spin-decomposed total density of states (TDOS) of the B(Ca)TN, BN(Ca)T, and BNT(Fe). The blue and yellow areas represent the majority-spin and minority-spin states, respectively. The Fermi level is set to zero energy.

We now explore the enhanced ferromagnetism of the Bi_0.5_Na_0.5_TiO_3_ with the Bi- [denoted B(Ca)NT] and Na-site Ca [BN(Ca)T], and Ti-site Fe substitutional dopant [BNT(Fe)]. Figure 10(a–c) show the 6 f.u. model structures of the B(Ca)NT, BN(Ca)T, and BNT(Fe) compounds, where only one atomic site was replaced by the substitutional dopant atom. We have also considered the 24 f.u. cell structures with a single-site doping but their atomic structures are not presented in the present paper. If one assumes full occupations of the Ca and Fe atom dopants in the host Bi_0.5_Na_0.5_TiO_3_, the highest experimental impurity concentration of 9 mol.% (as an example case) can be converted into (Bi_0.96_Ca_0.04_)_0.5_Na_0.5_TiO_3_ for Ca→Bi, Bi_0.5_(Na_0.6_Ca_0.4_)_0.5_TiO_3_ for Ca→Na, and Bi_0.5_Ca_0.5_Ti_0.9_Fe_0.1_O_3_ for Fe→Ti. In the computation, the doping concentration of 3.3 at.% (1 dopant per 30-atom cell) corresponds to [(Bi/Na)_0.67_Ca_0.33_]_0.5_(Na/Bi)_0.5_TiO_3_ for Ca→Bi/Na and Bi_0.5_Na_0.5_Ti_0.84_Fe_0.16_O_3_ for Fe→Ti, while it is [(Bi/Na)_0.92_Ca_0.08_]_0.5_(Na/Bi)_0.5_TiO_3_ for Ca→Bi/Na and Bi_0.5_Na_0.5_Ti_0.96_Fe_0.04_O_3_ for Fe→Ti for 0.83 at.% doping (1 dopant per 120-atom cell). Thus, we believe that the amount of the impurity defects in the experimental sample is somehow reflected in the present calculations. The TDOS of the B(Ca)NT, BN(Ca)T, and BNT(Fe) systems are shown in Fig. 11. The B(Ca)NT and BN(Ca)T exhibits nonmagnetic features, whereas there is a significant midgap state around the Fermi level for the BNT(Fe). In particular, such a midgap state is nondegenerate in the spin subbands, indicating the strong ferromagnetic nature.

**Figure 12:**
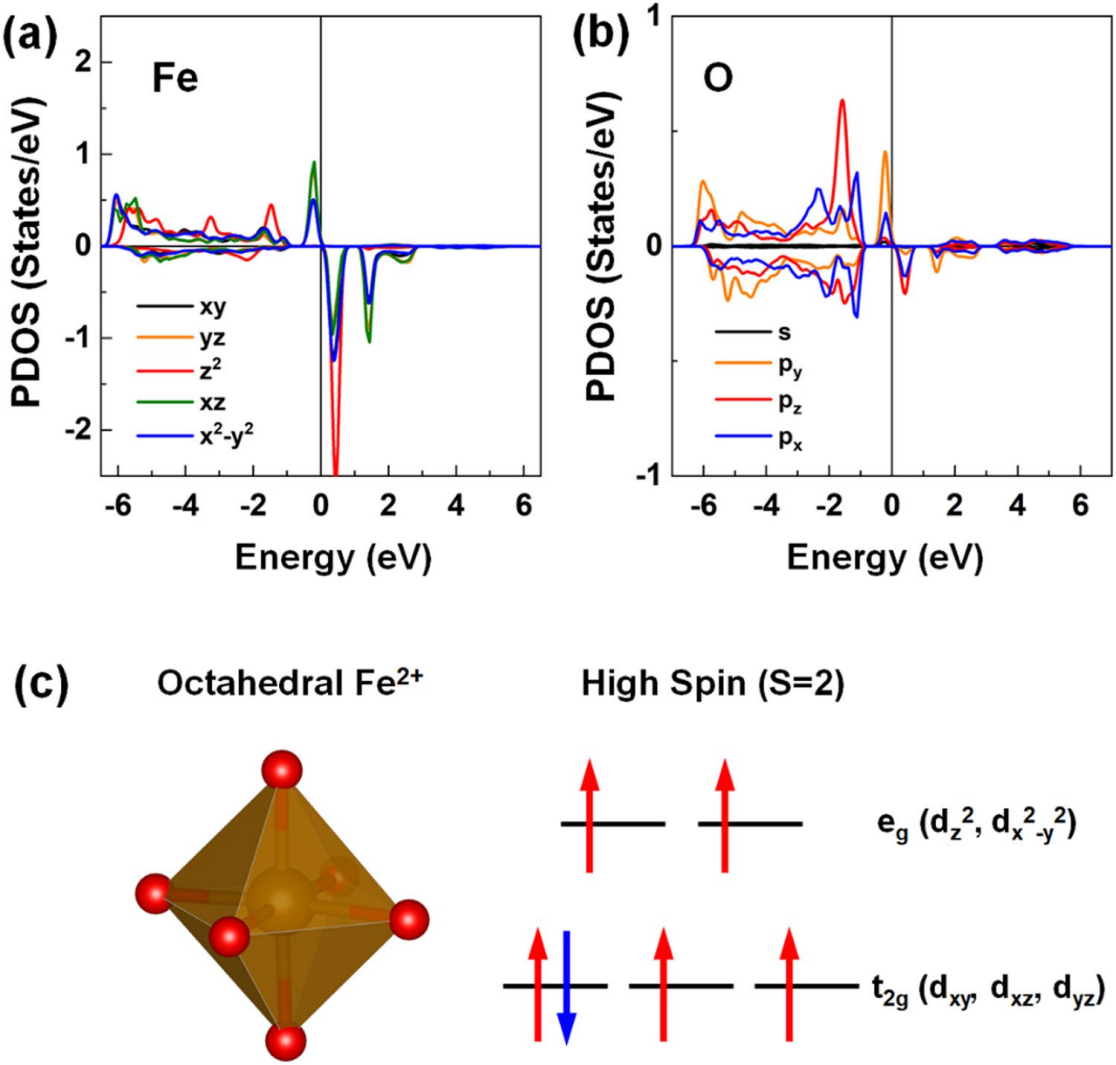
The PDOSs of the (**a**) *d*-orbital states of the Fe and (**b**) *s*- and *p*-orbital states of the O atom for the BNT(Fe). In (**a**), the black, orange, red, green, and blue lines represent the *d*_*xy*_, *d*_*yz*_, *d*_*z*_^2^, *d*_*xz*_, and *d*_*x*_^2^_–*y*_^2^ orbital states, respectively. In (**b**), the black, orange, red, and blue lines represent the *s*, *p*_*y*_, *p*_*z*_, and *p*_*x*_ orbital states, respectively. The Fermi level is set to zero energy. (**c**) Schematic representations of the octahedral environment of the Fe^2+^ ion (left) and its energy levels of *d*-orbital states with the high-spin-state crystal field (right). The larger orange and smaller red spheres represent the Fe and O atoms, respectively. The red upward and blue downward arrows denote the spin-up and spin-down electrons at the low-lying *t*_2g_ and high-lying *e*_g_ states, respectively.

To obtain more understanding, we show the *d*-orbital PDOS of the Fe atom of BNT(Fe) in Fig. 12(a). The corresponding *s*- and *p*-PDOS of the neighboring O atom is also shown in Fig. 12(b). Both the Fe and O provide the contribution to the midgap state. This indicates a strong orbital hybridization between the Fe 3*d* and O 2*p* states. In particular, the majority-spin bands of Fe were fully occupied, whereas the minority-spin states were partially unoccupied. Consequently, the Fe atom exhibited a substantially large exchange splitting between the spin sub-bands of the majority- and minority-spin states, resulting in a magnetic moment of approximately 4 µ_B_ per unit cell, which corresponds to 0.64 (0.16) µ_B_ for the 6 (24) f.u. cell structure. In Table 2, we present the calculated magnetization per f.u. cell (μ_B_/f.u.) of the B(Ca)NT, BN(Ca)T, and BNT(Fe) systems for the 6 and 24 f.u. structures. The induced moment at the neighboring O-site to the Fe was relatively minimal in the order of approximately 0.1 µ_B_.

**Table 2:** Magnetization per formula unit cell (μ_B_/f.u.) of Bi_0.5_Na_0.5_TiO_3_ with Bi and Na site Ca, and Ti site Fe substitutions for the 6 and 24 f.u. cells adopted in the DFT calculations.

Cell	B(Ca)NT	BN(Ca)T	BNT(Fe)
6-f.u	0.00	0.00	0.64
24-f.u	0.00	0.00	0.16

Based on the PDOS analyses, the schematic diagrams of the octahedral environment of Fe^2+^ ion (left) and its energy levels of the *d*-orbital states with the high-spin state crystal field (right) were produced, as shown in Fig. 12(c). The 6 *d*-orbitals of Fe^2+^ ion split by high-spin state according to crystal field theory were filled by the five majority-spin electrons in the low-lying *t*_2g_ orbital levels and by the electrons in the minority-spin *t*_2g_ state. Therefore, according to Hund’s rule, the calculated magnetic moment of 4 µ_B_ of the Fe replacement for the Ti-site can be explained by the electronic configuration of the high-spin state in crystal field theory through unpaired electron spin count. Furthermore, both *t*_2g_ and *e*_g_ states in PDOS were split slightly, due mainly to the Jahn–Teller effect because severe octahedron distortion occurred in the presence of the Ti-site Fe atoms. Mixed oxidation states of Fe^2+^ and Fe^3+^ might be possible in a practical situation if an O vacancy exists near the doping sites.

## Discussion

Lead-free ferroelectric Bi_0.5_Na_0.5_TiO_3_ materials are promising candidates for replacing for PZT-based materials in electronic devices because of requirement for environmental and human health protection. Recently, the discovery of room temperature ferromagnetism in intrinsic defects Bi_0.5_Na_0.5_TiO_3_ materials highlighted the potential to extend the function materials to smart electronic devices application. On the other hand, the magnetic performance of Bi_0.5_Na_0.5_TiO_3_ materials was lower such as magnetization which was usually less than 1 memu/g and of the diamagnetic component has a strong influence. Therefore, advancements in the magnetic performance properties of Bi_0.5_Na_0.5_TiO_3_ materials are required. In the present study, new solid solution of CaFeO_3-δ_-Bi_0.5_Na_0.5_TiO_3_ materials with greatly enhanced magnetic properties compared Bi_0.5_Na_0.5_TiO_3_ materials were fabricated. On the other hand, the substitution of Ca and Fe cations at the *A*-site and *B*-site, respectively, in perovskite Bi_0.5_Na_0.5_TiO_3_ materials, resulted in complex magnetic properties of the host materials. The origin of ferromagnetism in CaFeO_3-δ_-Bi_0.5_Na_0.5_TiO_3_ system was examined. The random incorporation of Fe cations at the Ti-site possibly induced ferromagnetism via super-exchange interaction of Fe cations through oxygen vacancies, such as Fe^3+^-□-Fe^3+^. The risk of Fe cations substitution in the host Bi_0.5_Na_0.5_TiO_3_ materials resulted in super-exchange between [Fe^3+^-□-Fe^3+^] versus [Fe^3+^-□-Fe^3+^] which normally favoured antiferromagnetic ordering. In addition, the isolated Fe cations distributed randomly into the host Bi_0.5_Na_0.5_TiO_3_ crystal exhibited paramagnetic behaviour. Thus, the complex magnetic properties of Bi_0.5_Na_0.5_TiO_3_ materials possibly tuned by varying the concentration of CaFeO_3-δ_ as a solid solution. However, unlike single Fe dopants, the presence of Ca cations into the host lattice exhibited complex results where both Ca^2+^ substitution for Bi^3+^ and Ca^2+^ substitution for Na^+^ cations produced the oxygen vacancies. The influence of intrinsic defects, including Bi, Na, Ti, and O vacancies on the electronic band structure was examined using DFT calculation to determine the contribution of intrinsic defects to the magnetic properties of the host Bi_0.5_Na_0.5_TiO_3_ materials. It was also predicted that the presences of Ti^3+^ and Ti^2+^ valence states could produce an intrinsic magnetism in the sample. In addition, a replacement of Ca for the Bi and Na sites and Fe for the Ti site was also clarified by the DFT calculations. We attribute the origin of weak ferromagnetism in pure Bi_0.5_Na_0.5_TiO_3_ mainly to the presence of the intrinsic defects. The theoretical prediction also indicates that the Bi and Na vacancies may induce a significant magnetic moment than that of oxygen vacancies. Indeed, these intrinsic defects in turn result in net magnetic moment for their neighbour oxygen sites. We suggest that the controlled valence state of transition metal defects was important for achieving optical magnetic moments in current integration ferromagnetic in lead-free ferroelectric materials. In other words, the co-modification of the *A*-site via alkali materials and *B*-site via transition metals were important parameters for estimating the increasing magnetic performance of lead-free ferroelectric materials. This study opens a new way to estimate the enhancement of the magnetic performance of lead-free ferroelectric materials via using the solid solution method in the current development of green multi-ferroics functional materials. In addition, this work not only applied to lead-free ferroelectric Bi_0.5_Na_0.5_TiO_3_-based materials, but may also be extended to lead-free ferroelectric BaTiO_3_-based, (Ba,Ca)(Ti,Zr)O_3_-based, or (K,Na)NbO_3_-based materials etc.

## Methods

### Sample preparation

The CaFeO_3-*δ*_ solid solution was prepared in Bi_0.5_Na_0.5_TiO_3_ materials via sol–gel method to obtain (1-*x*)BNT-*x*CaFeO_3-*δ*_ (*x* = 0, 0.5, 1, 3, 5, 7, and 9 mol.%). Raw materials such as Bi(NO_3_)_3_.5H_2_O, Ca(NO_3_)_2_, NaNO_3_, and Fe(NO_3_)_3_.9H_2_O were weighed and dissolved in deionized water and acetic acid. Subsequently, acetylacetone (CH_3_COCH_2_COCH_3_) was added to avoid hydrolysis. Tetraisopropoxytitanium (IV) (C_12_H_28_O_4_Ti) was also added and stirred continuously until it became transparent. The gels were prepared by drying the solution at 100 °C. The powdered samples were fabricated by sintering at 900 °C for 3 h in air.

### Sample characterization

The chemical composition of the samples was analyzed by energy-dispersive spectroscopy (EDS). Fig. S1(a,b) are EDS spectra of selected area for pure Bi_0.5_Na_0.5_TiO_3_ and CaFeO_3-δ_-modified Bi_0.5_Na_0.5_TiO_3_ materials with 5 mol.% CaFeO_3-δ_ as solid solution, respectively. The amount of sodium added was approximately 30 mol.% to compensate for the Na loss during the gelling and sintering processes after evaluation by electron probe microanalysis (EPMA)^35,36^. The valence state of Fe 2*p* cations in CaFeO_3-δ_-modified Bi_0.5_Na_0.5_TiO_3_ materials were measured by X-ray photoelectron spectroscopy (XPS), as shown for an example in Fig. S2. The crystal structure quality and vibrational modes of the powdered samples were determined by XRD and Raman spectroscopy, respectively. The absorbance of the samples was measured by ultraviolet–visible spectroscopy (UV-Vis). The PL spectra were recorded using a laser at excitation wavelength of 475 nm, and the magnetization at room temperature was measured using a vibrating sample magnetometer

### Electronic band structural calculations

To understand the observed magnetic and electronic properties, DFT calculations were performed within the projected augmented wave method^62^, as implemented in the Vienna *ab initio* simulation package (VASP)^63,64^. The generalized gradient approximation (GGA) formulated by Perdew, Burke, and Ernzerhof (PBE) was used to describe the electron exchange correlation potential^65^. An energy cutoff value of 500 eV was used for the plane-wave basis and a *k*-point mesh of 8 × 8 × 8 (5 × 5 × 5) for the 6 (24) f.u. cell of Bi_0.5_Na_0.5_TiO_3_ for the Brillouin zone (BZ) integration. To obtain optimized atomic structures, the atomic positions and lattice parameters were allowed to be fully relaxed until the largest force became less than 10^−2^ eV*/*Å and the change in the total energy between the two ionic relaxation steps was smaller than 10^−5^ eV.

## Supplementary information

Supplemental data.
