# Supplementary material for: Intrinsic and tunable ferromagnetism in Bi0.5Na0.5TiO3 through CaFeO3-δ modification
Source: Sci Rep. 2020 Apr 10;10:6189. doi: 10.1038/s41598-020-62889-w (PMC7148307; doi:10.1038/s41598-020-62889-w)
Supplement: Supplementary file 1 — Supplemental data. [file 41598_2020_62889_MOESM1_ESM.doc]

**Supporting information**

**Intrinsic and tunable ferromagnetism in Bi_0.5_Na_0.5_TiO_3_ through CaFeO_3-δ_ modification**

N. T. Hung^1,2^, N. H. Lam^1^, N. A. Duc^2,3^, L. H. Bac^1^, N. N. Trung^1^, D. D. Dung^1,*^, Y. S. Kim^3,ξ^, N. Tsogbadrakh^4^, T. Ochirkhuyag^5^, and D. Odkhuu^5,+^

^1^School of Engineering Physics, Ha Noi University of Science and Technology,

1 Dai Co Viet road, Ha Noi, Viet Nam

^2^Department of Physics, Faculty of Basic-Fundamental Sciences, Viet Nam Maritime University, 484 Lach Tray Road, Le Chan, Hai Phong, Viet Nam

^3^Department of Physics, University of Ulsan, Ulsan 680-749, Republic of Korea

^4^Department of Physics, National University of Mongolia, Ulaanbaatar 14201, Mongolia

^5^Department of Physics, Incheon National University, Incheon 22012, Republic of Korea

Corresponding author:

D. D. Dung, e-mail: [dung.dangduc@hust.edu.vn](mailto:dung.dangduc@hust.edu.vn),

Y. S. Kim, e-mail: [yskim2@ulsan.ac.kr](mailto:yskim2@ulsan.ac.kr),

D. Odkhuu, e-mail: [odkhuu@inu.ac.kr](mailto:odkhuu@inu.ac.kr)

**1. Chemical compositions.**

**
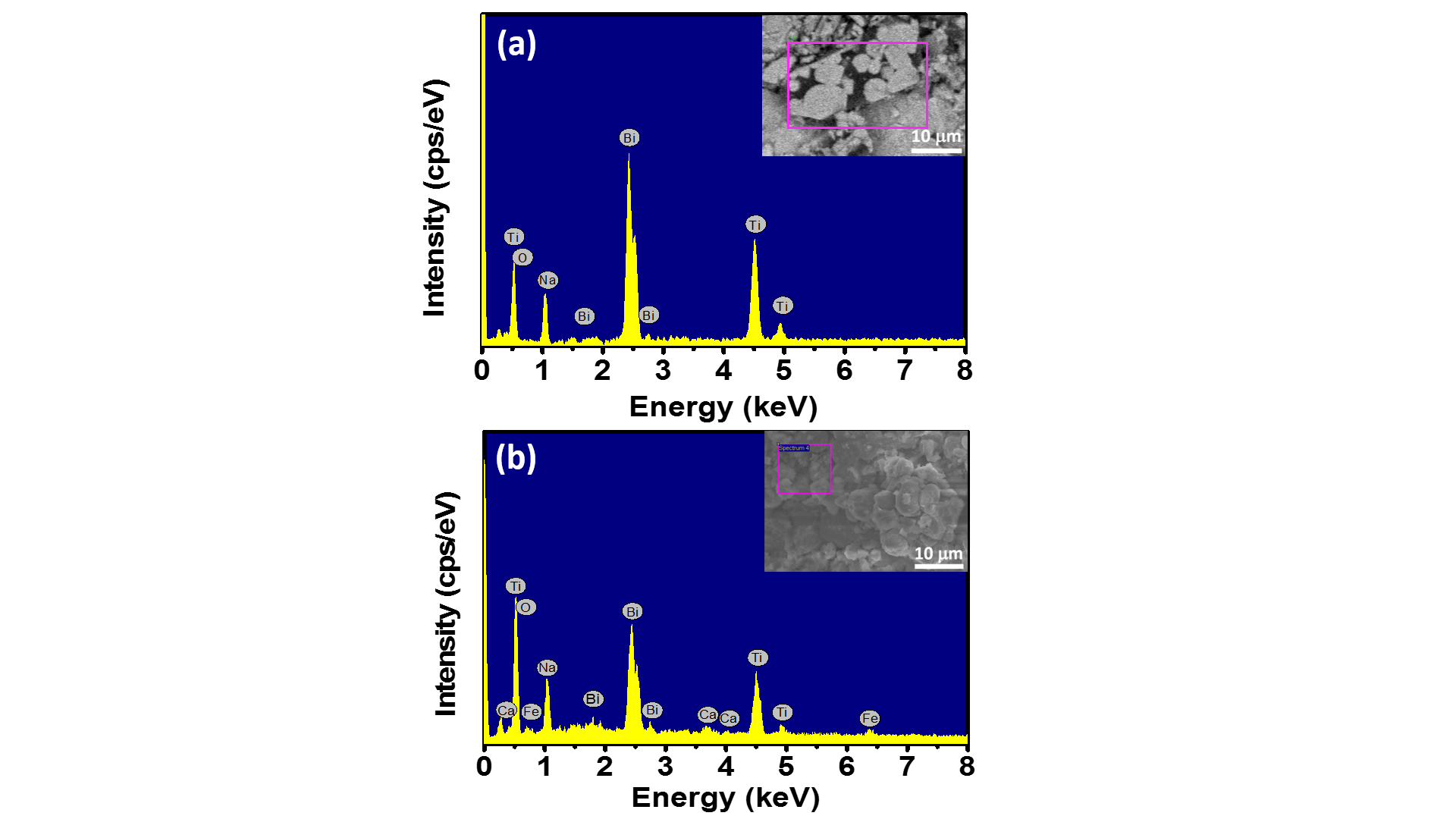
**

**Fig. S1.** EDS spectra of (a) pure Bi_0.5_Na_0.5_TiO_3_ materials and (b) CaFeO_3-_*_δ_*-modified Bi_0.5_Na_0.5_TiO_3_ materials with 5 mol% CaFeO_3-δ_ as solid solution. The inset of each figure indicates the selected area for composition characterization.

**2. Valence state of Fe cations.**


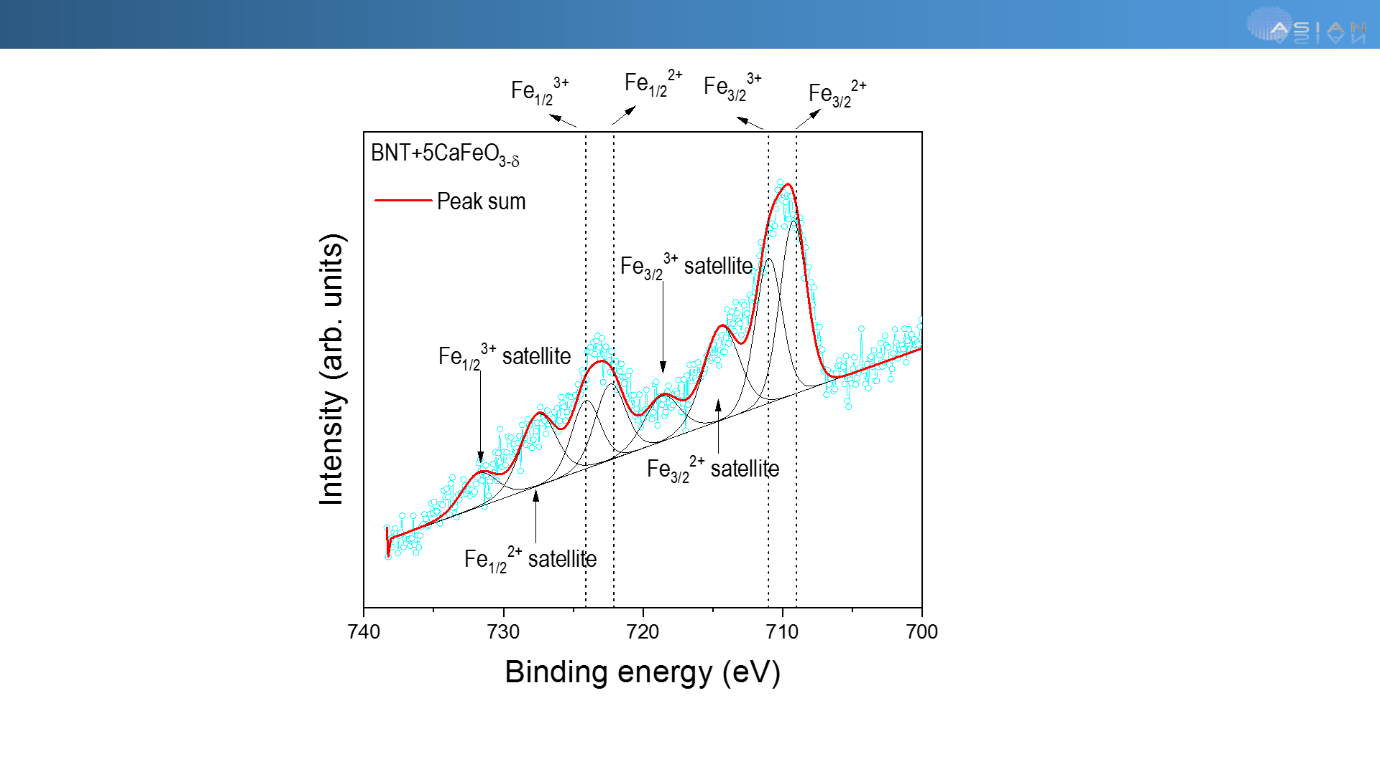


**Fig. S2.** XPS spectra of Fe 2*p* for selected CaFeO_3-δ_-modified Bi_0.5_Na_0.5_TiO_3_ with 5 mol.% as solid soluditon.

**3. Electronic band structure.**


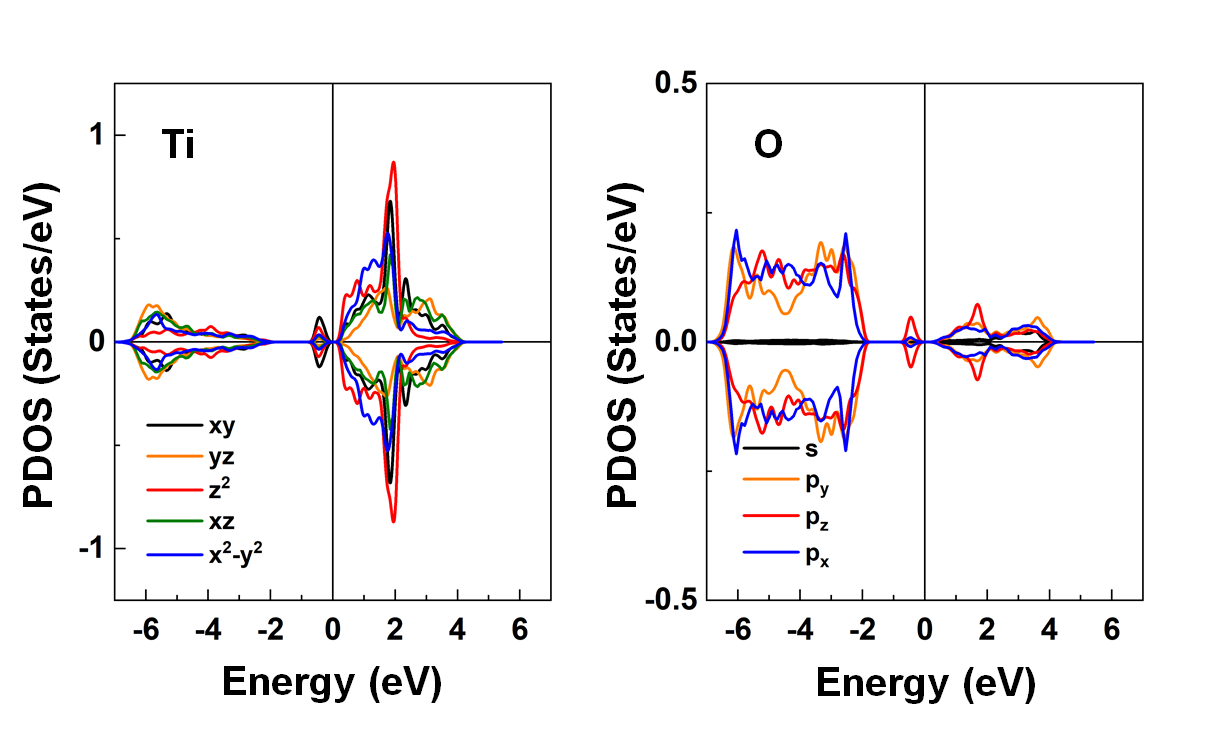


**Fig. S3.** The *d*-orbital decomposed PDOS of the Ti atom (left) and the *s*- and *p*-orbital PDOS of the O atom neighboring to an O-vacancy of the BTN(V_O_) compound. The black, orange, red, green, and blue lines represent the *d_xy_*, *d_yz_*, *d_z_*^2^, *d_xz_*, and *d_x_*^2^–*_y_*^2^ orbital states, respectively. The black, orange, red, and blue lines represent the *s*, *p_y_*, *p_z_*, and *p_x_* orbital states, respectively. The Fermi level is set to zero energy.
